# Stratification of adolescents across mental phenomena emphasizes the importance of transdiagnostic distress: a replication in two general population cohorts

**DOI:** 10.1007/s00787-021-01909-0

**Published:** 2021-11-18

**Authors:** Jan Stochl, Hannah Jones, Emma Soneson, Adam P. Wagner, Golam M. Khandaker, Stanley Zammit, Jon Heron, Gemma Hammerton, Edward T. Bullmore, Ray Dolan, Peter Fonagy, Ian M. Goodyer, J. Perez, Peter B. Jones

**Affiliations:** 1 Department of Psychiatry, Herchel Smith Building for Brain and Mind Sciences, University of Cambridge, Cambridge Biomedical Campus, Cambridge, CB2 0SZ UK; 2 National Institute for Health Research Applied Research Collaboration East of England, Cambridge, UK; 3 Department of Kinanthropology, Charles University, Prague, Czechia; 4 Centre for Academic Mental Health, Bristol Medical School, University of Bristol, Bristol, UK; 5 Norwich Medical School, University of East Anglia, Norwich, UK; 6 Cambridgeshire and Peterborough NHS Foundation Trust, Cambridge, UK; 7 MRC Centre for Neuropsychiatric Genetics and Genomics, Cardiff University School of Medicine, Cardiff, UK; 8 Wellcome Trust Centre for Neuroimaging, University College London, London, UK; 9 Department of Clinical, Educational and Health Psychology, University College London, London, UK; 10 Institute of Biomedical Research of Salamanca (IBSAL), Psychiatry Unit, Department of Medicine, University of Salamanca, Salamanca, Spain

**Keywords:** Psychopathology, Classification, Adolescence, Transdiagnostic approach

## Abstract

Characterizing patterns of mental phenomena in epidemiological studies of adolescents can provide insight into the latent organization of psychiatric disorders. This avoids the biases of chronicity and selection inherent in clinical samples, guides models of shared aetiology within psychiatric disorders and informs the development and implementation of interventions. We applied Gaussian mixture modelling to measures of mental phenomena from two general population cohorts: the Avon Longitudinal Study of Parents and Children (ALSPAC, *n* = 3018) and the Neuroscience in Psychiatry Network (NSPN, *n* = 2023). We defined classes according to their patterns of both positive (e.g. wellbeing and self-esteem) and negative (e.g. depression, anxiety, and psychotic experiences) phenomena. Subsequently, we characterized classes by considering the distribution of diagnoses and sex split across classes. Four well-separated classes were identified within each cohort. Classes primarily differed by overall severity of transdiagnostic distress rather than particular patterns of phenomena akin to diagnoses. Further, as overall severity of distress increased, so did within-class variability, the proportion of individuals with operational psychiatric diagnoses. These results suggest that classes of mental phenomena in the general population of adolescents may not be the same as those found in clinical samples. Classes differentiated only by overall severity support the existence of a general, transdiagnostic mental distress factor and have important implications for intervention.

**Supplementary Information:**

The online version contains supplementary material available at 10.1007/s00787-021-01909-0.

## Introduction

Most psychiatric disorders begin in adolescence and early adulthood [1–3]. While the prevalence of conventionally defined disorders in the adolescent population is well-known [1], the patterning of mental phenomena in the general population has received less attention. Historically, psychiatric diagnosis has paralleled medical diagnosis: sets of symptoms are used to identify aetiologies and courses of illness as well as treatment options. Psychiatric classification systems are operationalised through Diagnostic and Statistical Manual of Mental Disorders (DSM) and International Classification of Diseases (ICD) criteria, which were developed largely by expert consensus rather than empirical findings [4]. The validity of these classifications is increasingly challenged by recent neurobiological and genetic evidence that often transcends diagnostic boundaries [5] and evidence of high comorbidity and longitudinal shifts in disorder diagnosis over the lifecourse [6]. This accords with the well-established heterogeneity of treatment outcomes for the same psychotherapeutic or pharmacological interventions used in people with the same diagnosis [7]. As such, the usefulness of diagnostic categories remains uncertain [8, 9].

In response to the limitations of traditional psychiatric diagnosis, ‘transdiagnostic’ approaches to psychiatry have gained some traction [10]. Transdiagnostic approaches cut across diagnostic categories or move beyond them entirely [11] and represent a significant paradigm shift away from the traditional diagnostic model [10]. Proponents of transdiagnostic approaches argue for several advantages of the model, including that it ‘opens up new ways of conceptualizing the underlying theories and processes implicated in mental ill health and provides a platform for novel ways of thinking about onset, maintenance, and clinical treatment and recovery from experiences of disabling mental distress’ [11]. Transdiagnostic models are also supported by empirical findings showing that a single latent variable, often called ‘general mental distress’ or ‘the p-factor’, underlies diagnoses [12–18].

Analytically-sound, ‘bottom-up’ classification approaches using a comprehensive range of both negative phenomena (i.e. psychiatric symptoms) and positive phenomena (e.g. mental wellbeing) can greatly contribute to the debate surrounding psychiatric nosology and its applications [4]. Characterizing patterns of mental phenomena in population-based studies of young people can provide insight into the latent organization and concomitant burden of psychiatric disorders whilst avoiding the biases of chronicity and selection inherent in variously collected clinical samples. Such characterization can also guide development of models of the phenotypic organization and shared aetiology of psychiatric disorders [19]. Clinically, clarity on the patterning of mental phenomena could be useful for determining whether interventions should be guided by diagnostic classification, levels of transdiagnostic distress or both. This may be particularly appropriate for young people whose presenting symptoms do not fit any particular diagnostic category [20], or those with high comorbidity. So-called ‘soft’ analytical approaches [such as such as latent class analysis (LCA) or Gaussian finite mixture models (GMMs)], which allocate individuals into classes stochastically, are required to investigate these alternatives, avoiding the assumptions inherent in ‘hard’ methods (such as k-means) that ignore measurement error [21].

Our aim was to explore how adolescents from the general population can be classified into homogeneous classes characterized with respect to patterns of positive and negative mental phenomena. We hypothesized that classes would mirror diagnostic categories (i.e. class of individuals with predominantly depressive symptoms, another class with predominant anxiety symptoms, etc.). We then characterized the derived classifications by considering the distribution of diagnoses and sex split across classes.We aimed to be broad in our consideration of a range of symptoms related to mental health including positive phenomena such as mental wellbeing or self-esteem [1].

## Material and methods

### Participants

#### Cohort 1: the Neuroscience in Psychiatry Network (NSPN 2400) cohort

The NSPN 2400 cohort (www.nspn.org.uk) [22] comprises 2403 voluntary participants aged 14–25 recruited from Cambridgeshire and London. The main sampling framework comprised age-sex registers of 41 general practices (primary health care practices) recruited through the National Institute for Health Research (NIHR) Clinical Research Network, and 19 secondary schools/colleges. Through the practices and schools, study invitations were sent in batches to support recruitment of 200 young women and men into five age strata. Nearly a fifth of participants (18.3%) were recruited through the study website.

Here, we used a subsample of *n* = 2023 subjects with non-missing data on all measures. This subsample has a mean age of 19.1 years [standard deviation (SD) = 3.0 years] and is 53.4% (1071) female. Additionally, most participants had a recorded ethnicity of ‘white’ (79.4%) and over half (61%) were recruited in Cambridgeshire.

#### Cohort 2: the Avon Longitudinal Study of Parents and Children (ALSPAC)

The “core” enrolled sample consisted of 14541 pregnant women residing in the former county of Avon, United Kingdom, who had an expected date of delivery between 1st April 1991 and 31st December 1992. When the oldest children were approximately 7 years of age, an attempt was made to bolster the initial sample with eligible cases who had failed to join the study originally. The total sample size for analyses using any data collected after the age of seven is therefore 15247 pregnancies, resulting in 15458 foetuses. Of this total sample of 15458 foetuses, 14775 were live births and 14,701 were alive at 1 year of age. Parents and children have been followed up regularly since recruitment via questionnaire and clinic assessments. Further details on the sample characteristics and methodology have been described previously [23, 24] and detailed information about ALSPAC can be found on the study website (http://www.bristol.ac.uk/alspac). For information on all available ALSPAC data see the fully searchable data dictionary (http://www.bristol.ac.uk/alspac/researchers/our-data/). Ethical approval for this study was obtained from the ALSPAC Ethics and Law Committee and the Research Ethics Committees.

Only the 3018 participants (1253 males and 1765 females) with a mean age of 16.5 years with complete data were considered here. The majority of participants were white (2693; 89.2%), 104 (3.4%) were non-white and ethnicity was unknown for 221 (7.3%) participants. Sensitivity analysis carried out by Jones et al. [25] revealed this subsample was generally representative of the whole sample (which itself was representative of UK population).

### Measures

We aimed to be as broad as possible in our consideration of a wide range of mental phenomena [1]. Negative phenomena included measures of depression [33-item Moods and Feelings Questionnaire (MFQ) [26] in NSPN and its short, 13-item version (SMFQ) [27] in ALSPAC], anxiety [28-item Revised Children’s Manifest Anxiety Scale (RCMAS) [28] and a selection of items from the Development and Well Being Assessment (DAWBA) [29]], obsessive–compulsive disorder [11-item Revised Leyton Obsessional Inventory (LOI) [30]], psychotic experiences [10 items from the Psychosis-Like Symptoms Questionnaire (PLIKS-Q) [31]], negative psychotic symptoms [11 negative symptoms from the Community Assessment of Psychic Experiences (CAPE) [32]], and antisocial behaviour [11-item Antisocial Behaviour Questionnaire (ABQ)]. Positive phenomena included measures of self-esteem [10-item Rosenberg Self-Esteem Scale (RSES) [33]] and psychological wellbeing [14-item Warwick-Edinburgh Mental Wellbeing Scale (WEMWBS) [34]]. We included these positive phenomena in addition to the negative phenomena because previous research has shown that they can be informative in regard to characterising psychopathology [13]. It is important to stress that we used information from individual measure items as input data for our analyses (i.e. not summary scores from the measures). In total, 107 items were used as indicators of classes for NSPN and 66 items for ALSPAC. See TableS1 in Online Resource 1 for an overview of extracted measures and their descriptive statistics. Additional variables, primarily used for characterisation, included sex and diagnoses [self-reported in NSPN or based on clinically-oriented interviews (The Clinical Interview Schedule-Revised (CIS-R) and PLIKS-i) in ALSPAC].

### Analysis

#### Number of classes

We first explored the optimal number of classes of individuals with similar patterns of mental phenomena using Gaussian finite mixture modelling (GMM). This ‘soft’ clustering algorithm has a number of advantages over traditional approaches to classification such as latent class analysis (LCA) when there is a large number of indicator variables [35], such as in this study. GMM aims to find a small number of homogeneous classes with respect to patterns in observed item-level data through decomposition of complex distributions in multivariate space into small number of Gaussian distributions. Since finding an analytical solution to maximisation of the log likelihood for a GMM is not possible, an iterative approach is required. The expectation maximisation (EM) algorithm is well-suited to this problem. The GMM with EM algorithm is available in R [36] within the mclust [37, 38] package. Using this package, we fitted various models differing in the number of classes and cluster shape [38] using individual items. Evaluations of model fit and determination of the optimal number of classes were based on the Bayesian information criterion (BIC) [39], which provides superior performance in classes enumeration over other indices such as Akaike information criterion (AIC) [40, 41].

#### Model parsimony

While the BIC often supports reasonable selection of the appropriate number of Gaussian distributions in the mixture distribution, the number of classess is often fewer. Indeed, a class may have a non-Gaussian distribution. Consequently, a class may be better represented by merging two or more Gaussian distributions in the mixture. The integrated classification likelihood (ICL) [42] was conceived as an alternative for determining the number of classes (which typically favours more parsimonious solutions) rather than the number of mixture components. Finally, we considered the parsimony of our model using the methodology proposed by Baudry et al. [43] which proposes a combination of mixture components according to an entropy criterion.

#### Multidimensional scaling

To visualize classes, we used multi-dimensional scaling (MDS), which ‘projects’ higher dimensional data onto fewer dimensions. Here, MDS shows the distances between any two individuals in a three-dimensional (3D) plot. Given the ordinal nature of the data, the distance matrix was calculated using formulae by Gower [44] implemented within the *cluster* package [45]. MDS is conducted on the resulting matrix using the *sna* package [46]. This calculates the (projected) coordinates in 3D space, visualized using the *plotly* package [47].

#### Chracterizing the classes

We examined the classes according to standardized scores derived from our extracted measures. Subsequently, we characterized classes by considering the distribution of diagnoses and sex split across classes. A chi-square test was used to compare whether the proportion of men and women differed between classes.

## Results

### Determining the optimum number of classes (Gaussian mixture modelling)

#### Model fit

**Fig. 1:**
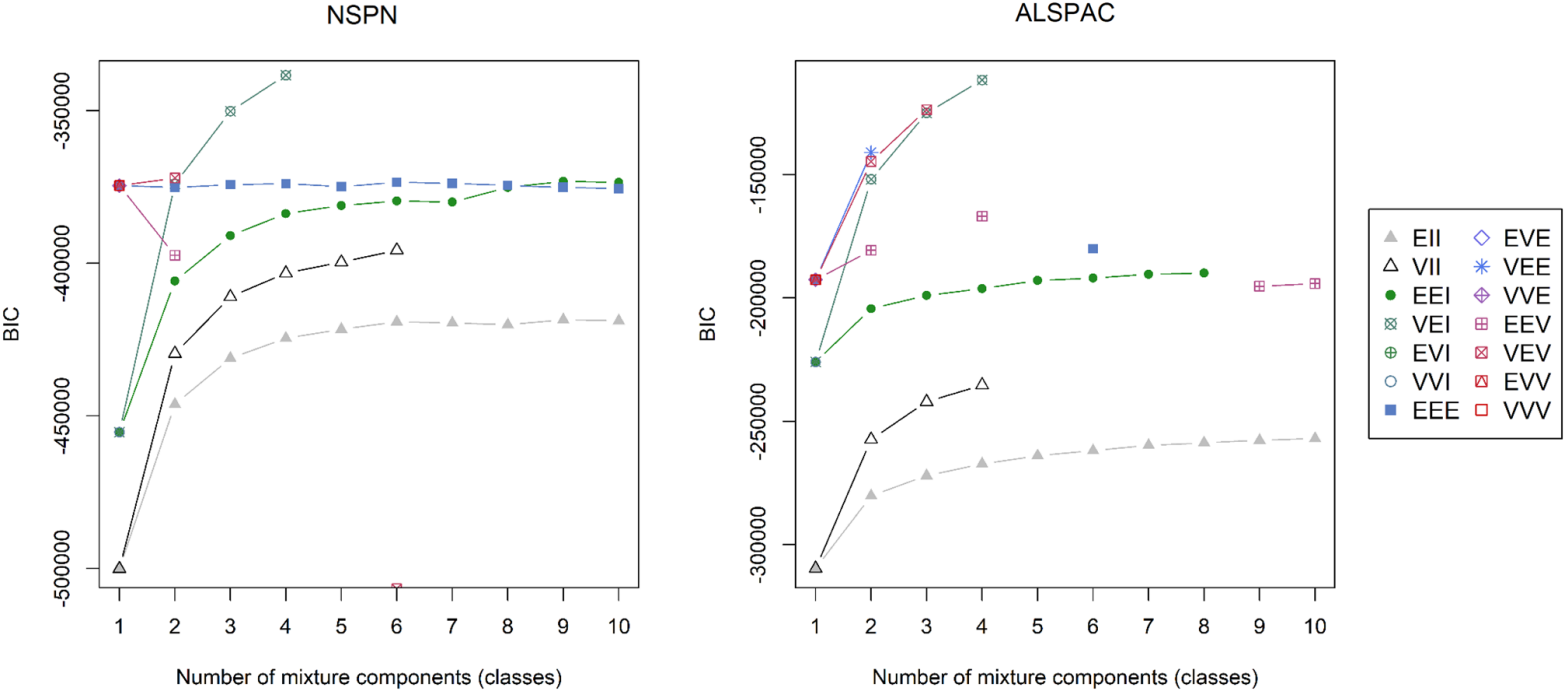
BICs of fitted Gaussian mixture models of different shapes and number of classes for NSPN (left) and ALSPAC (right). Missing points for some models indicate that the specific model did not converge. Model acronyms are explained in Table 1 and detailed in [38, 48]

The fit, as measured by the BIC, for a variety of finite mixture models differing in number of classes (or, more precisely, mixture components), and shape and orientation of mixture classes are shown in Fig. 1 (an explanation of model acronyms is provided in Table 1, and a detailed schematic is provided in Scrucca et al. [48]). Here, higher BICs indicate more parsimonious models.

**Table 1:** Explanation of models acronyms

Model	Distribution	Volume	Shape	Orientation
EII	Spherical	Equal	Equal	Not applicable
VII	Spherical	Variable	Equal	Not applicable
EEI	Diagonal	Equal	Equal	Coordinate axes
VEI	Diagonal	Variable	Equal	Coordinate axes
EVI	Diagonal	Equal	Variable	Coordinate axes
VVI	Diagonal	Variable	Variable	Coordinate axes
EEE	Ellipsoidal	Equal	Equal	Equal
EVE	Ellipsoidal	Equal	Variable	Equal
VEE	Ellipsoidal	Variable	Equal	Equal
VVE	Ellipsoidal	Variable	Variable	Equal
EEV	Ellipsoidal	Equal	Equal	Variable
VEV	Ellipsoidal	Variable	Equal	Variable
EVV	Ellipsoidal	Equal	Variable	Variable
VVV	Ellipsoidal	Variable	Variable	Variable

For both cohorts, the optimal models were those comprising four classes that were diagonal with variable volume but of equal shape (a VEI model). In both cohorts, and in accordance with BIC, ICL (see FigS1 in Online Resource 1) indicated a VEI with four mixture components as the best fitting model.

#### Distribution of individuals across the classes

In this model, each individual has a calculated probability of being in each class, with final allocation determined by the greatest probability. Allocation uncertainty, an important consideration in any classification model, was low in the four-class VEI models. In NSPN, 96% (1939/2023) of individuals were allocated into classes with probabilities larger than 0.95 and only 12 were allocated with probabilities less than 0.60. In ALSPAC, 96% (2895/3018) were allocated with probabilities of 0.95 or higher and only 14 were allocated with probabilities less than 0.60. This suggests good class separation.

Another desirable quality is for no class to contain only a small proportion of members: small classes can be difficult to interpret and can indicate convergence issues. Table 2 indicates a reasonable distribution of individuals across all classes.

**Table 2:** Distribution of individuals across classes

	Low	Low-medium	Medium–high	High
NSPN (*n* = 2023)	429 (21.2%)	774 (38.3%)	622 (30.7%)	198 (9.8%)
ALSPAC (*n* = 3018)	552 (18.3%)	1073 (35.6%)	1065 (35.3%)	328 (10.9%)

### Parsimony considerations and class merging

**Fig. 2:**
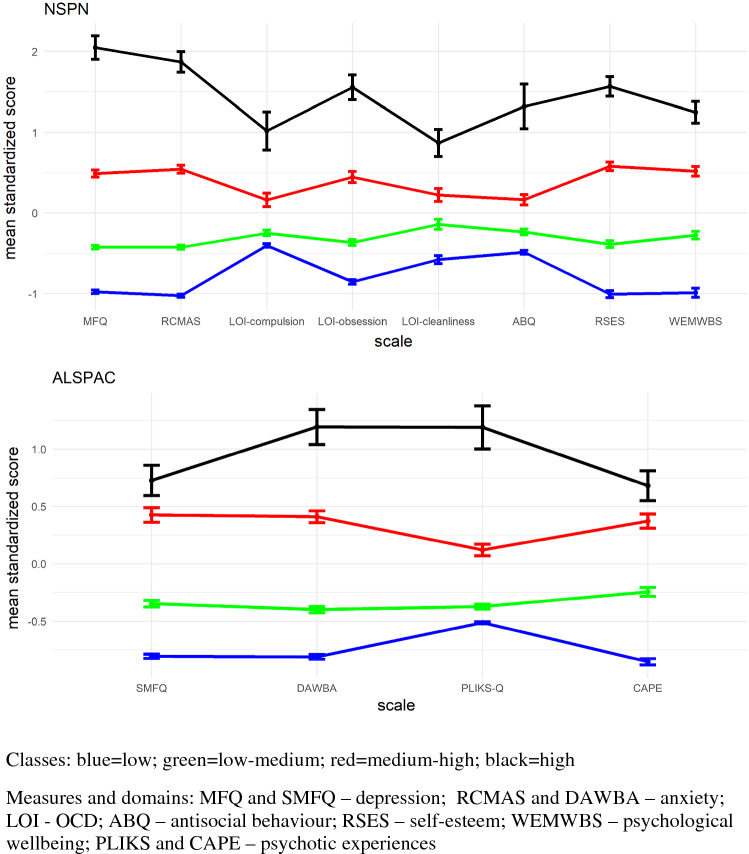
Standardized scale scores and 95% confidence intervals across classes for NSPN (top) and ALSPAC (bottom). Scoring of RSES and WEMWBS was reversed to match direction of other measures

The procedure developed by Baudry et al. [43] highlights whether some classes should be merged. A sequence of different classifications is built, starting from a well-fitting mixture model. The number of components in this base model is determined using the BIC. Next, from the base model, successive pairs of mixture components are merged such that chosen pairs minimize the entropy (measure of classification uncertainty with lower values indicating less ambiguous class allocation). FigS2 in Online Resource 1 shows how entropy varies with the number of classes in the VEI model. It indicates that the trends in the entropy values are, marginally, non-linear. Further, among the piecewise regression lines fitted, the ‘elbows’ are not particularly distinct, meaning that merging classes is not warranted [43].

### Visualizing the classes using multi-dimensional scaling (MDS)

**Fig. 3:**
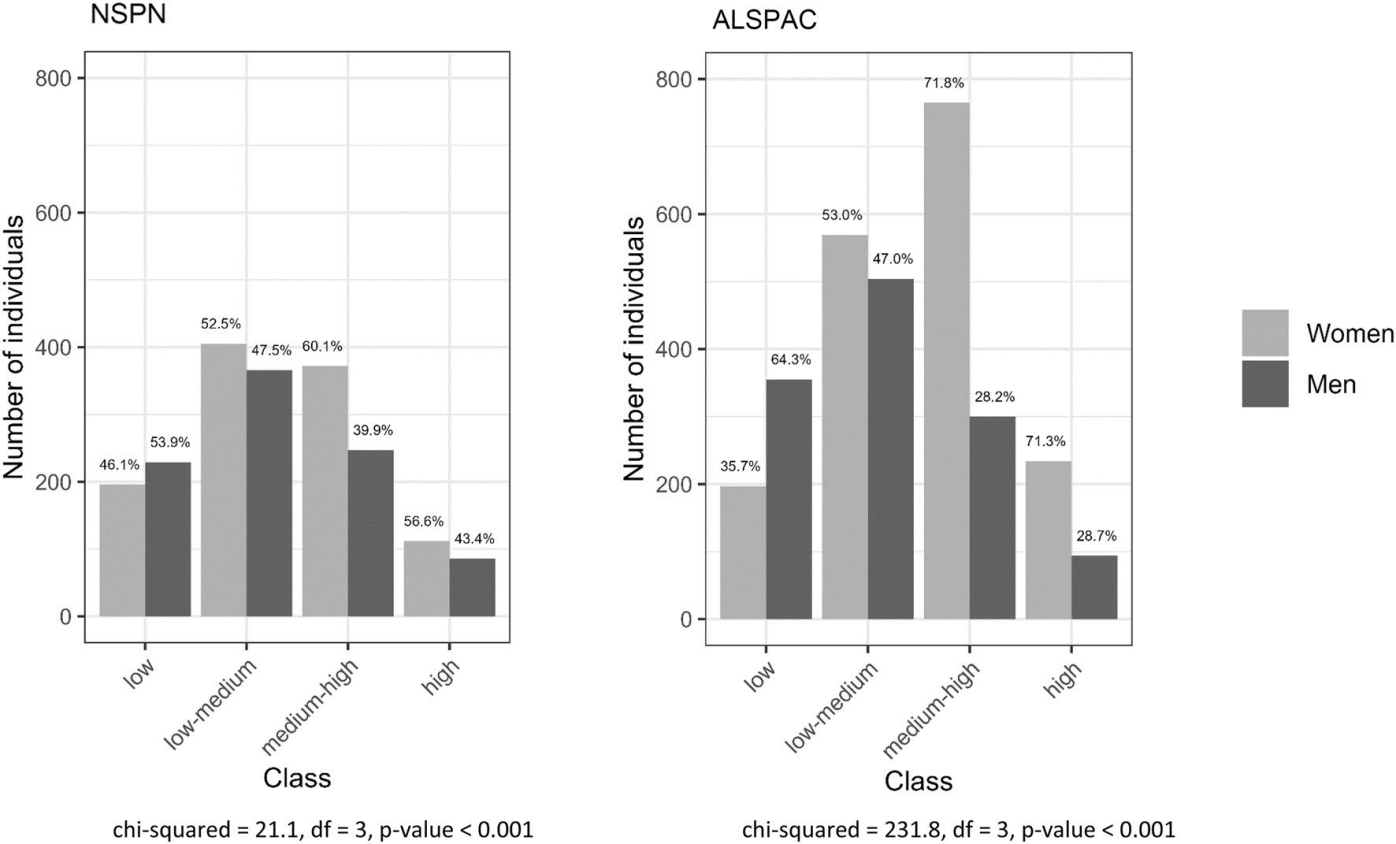
Distribution of sex across classes within each cohort (NSPN left; ALSPAC right)

FigS3 in Online Resource 1 shows a simplified representation of the fitted model structure within each cohort generated through MDS. Interactive versions of these plots are available at https://tinyurl.com/y6mclowk and https://tinyurl.com/y2964jgh. It is important to note these plots’ limitations: since classification was performed in high dimensional space, classes may not be appear distinct in these simplified three-dimensional plots.

Broadly, the NSPN cohort shows greater variability along the *x*-axis and clearer separation of classes, with much less heterogeneity along *y*- and *z-* axes. The ALSPAC cohort shows more homogenous variability along all three axes.

Classes differ in their levels of variability similarly across the two cohorts. For instance, the blue classes are relatively much more compact, while the black classes are much more dispersed, suggesting greater variability in the patterning of mental phenomena among class members.

### Characterizing the classes

To begin to characterize the classes, we considered their respective profiles of mental phenomena: Fig. 2 shows standardized scores and associated 95% confidence intervals (CIs) for various mental health measures.

The profiles in Fig. 2 show that classes differ primarily by severity of transdiagnostic distress, the latent construct underlying the severity of mental phenomena. Importantly, this suggests that adolescents within the general population primarily cluster by severity of distress rather than traditional clinical diagnostic categories. Broadly, variability increases with severity: the high- (black) and medium–high- (red) severity classes have the largest CIs, indicating the largest variability, while the low- (blue) and low–medium- (green) severity classes are more alike in terms of mental phenomena (this aligns with the observations on the 3D projections in Online Resource 1 FigS3). Individuals with more severe distress (i.e. high symptom scores and low wellbeing) also have a greater probability of rare psychopathological items such as psychotic experiences. Finally, there is small within- and large between-class variability, indicating good class separation.

For NSPN, the order of classes in terms of severity of distress matches the order seen in the 3D projection (see Online Resource 1 FigS3, top), suggesting that the *x*-axis (the first principal component of the distant matrix which explains the largest proportion of variance) approximately represents severity.

#### Sex difference across classes

Figure 3 shows the sex split within classes. Within both cohorts, as severity of distress increases across the classes, the proportion of women also increases (with exception of the high-severity class, where this proportion slightly drops compared with the medium–high-severity class). Proportions of men and women are significantly different between classes (*p* < 0.001) in both cohorts.

#### Distribution of diagnoses across classes

The distributions of diagnoses are reported in Table 3. The distributions provide evidence of validity for the proposed classes in terms of clinically-relevant information. For example, the high-severity classes have the highest proportion of diagnoses; this proportion reduces with severity levels from medium–high-, to low–medium-, and finally to low–severity classes. This pattern replicates across cohorts and conditions except for unspecified psychiatric conditions (NOS) in the NSPN cohort.

**Table 3:** Distribution of self-reported diagnoses (NSPN) and clinically rated diagnoses/psychopathology (ALSPAC) across classes

	NSPN				ALSPAC			
	Low (*N* = 429)	Low-medium (*N* = 774)	Medium–high (*N* = 622)	High (*N* = 198)	Low (*N* = 552)	Low-medium (*N* = 1073)	Medium–high (*N* = 1065)	High (*N* = 328)
With any diagnosis	5 (1.2%)	11 (1.4%)	42 (6.8%)	45 (22.7%)	14 (3%)^a^	63 (7%)^a^	168 (18.5%)^a^	82 (30.3%)^a^
Depression	3 (0.7%)	5 (0.6%)	29 (4.7%)	35 (17.7%)	6 (1.3%)	31 (3.7%)	92 (10.8%)	45 (17.5%)
Anxiety	0 (0%)	4 (0.5%)	4 (0.6%)	5 (2.5%)	9 (2%)	38 (4.5%)	105 (12.3%)	56 (21.8%)
Psychosis/schizophrenia	0 (0%)	0 (0%)	1 (0.2%)	1 (0.5%)				
Bipolar	0 (0%)	0 (0%)	0 (0%)	2 (1%)				
Obsessive–compulsive	0 (0%)	0 (0%)	0 (0%)	1 (0.5%)				
Autism	0 (0%)	1 (0.1%)	2 (0.3%)	1 (0.5%)				
Unspecified	2 (0.5%)	1 (0.1%)	6 (1%)	0 (0%)				
Psychotic experiences					1 (0.2%)	4 (0.5%)	12 (1.4%)	14 (5.3%)

^a^Some individuals had more than one diagnosis and that is why this number is not a sum of individuals with specific diagnoses

## Discussion

Whether, and how, the adolescent population can be classified into homogeneous classes with respect to mental phenomena are important epidemiological questions for informing the phenotypic organization and nosology of psychiatric disorders [19]. This study aimed to empirically explore the number of homogeneous classes in adolescents, using two large general population cohorts to allow us to replicate or refute the findings. The characterization of classes was guided by a variety of positive and negative mental phenomena. The classes were further characterized using external variables including sex and existing diagnoses.

The results were strikingly similar across cohorts. Four classes gave the most parsimonious distribution of individuals. These classes primarily differed by overall severity of transdiagnostic distress, rather than particular patterns of negative phenomena akin to diagnoses. However, this does not mean that transdiagnostic distress should be interpreted as linear (a purely linear interpretation would be warranted if only one class were extracted). A four-class solution suggests that severity is not linear but might be better represented in (four) ‘levels’, metaphorically reminiscent of energy-dependent electron orbits in quantum mechanics. This provides a new perspective to the extensive discussion about the dimensional vs. categorical nature of mental health disorders [49–52]; so that mental health disorders might be a mix of both. Further, these four classes differed with respect to within-class variability: with increasing overall severity between the four classes, the within-class phenomenological richness (variability) increased, too.

The cohorts had similar distributions of individuals across classes. The low-severity classes represented approximately one fifth of each cohort. These individuals scored consistently well across various measures of positive and negative phenomena, and few (1.2–3%) reported current or past diagnoses. This class had the lowest proportion of women. The low-medium-severity classes constituted just over a third of each cohort. Individuals in this class generally scored slightly better than average across the measures. Also representing approximately one third of each cohort, the medium–high–severity classes consisted of more severely distressed individuals. Individuals in these classes scored worse in terms of positive and negative phenomena and had proportionately more diagnoses than the low and low–medium–severity classes. The low–medium- and medium–high–severity classes had the largest proportions of women. Finally, the high-severity classes constituted around a tenth of each cohort. The variability in scores was larger within this class than other classes. This class had the highest prevalence of clinical (30.3% in ALSPAC) and self-reported (22.7% in NSPN) diagnoses, reflecting high levels of transdiagnostic distress.

Our findings have some similarities but also key differences compared with findings obtained through previous latent class analyses. These characterizations (focused primarily on negative phenomena) have suggested four [19, 53] or five [54] distinct classes, with four-class models assigning the majority of individuals (62.5–84.0%) to a class characterized by low severity and a small proportion of individuals to a class characterized by high severity (1.6–4.2%) [19, 53]. However, while others have proposed classes characterized by specific diagnoses (e.g. depression/anxiety), the replicable classification within our study did not reflect this. Rather, the classes differed by severity of transdiagnostic distress, indirectly supporting the existence of a general mental distress factor, as found in previous studies using distinct methodology [12–18]. These studies show that multiple diagnostic categories are part of a single continuum, mirroring a more general domain (termed ‘general psychological distress’, ‘general mental distress’, ‘common mental distress’ or ‘p-factor’) and that diagnostic categories represent severities along this continuum [14].

In the ongoing debate on the merits of diagnostic versus transdiagnostic approaches to psychiatry, our work is consistent with the idea of considering psychiatric presentations as part of a single dimension (akin to ‘general mental distress’ or ‘p-factor’) rather than strict categorical entities represented by diagnoses. However, consideration of distress does not feature strongly or consistently in current ICD or DSM diagnostic systems, which rely primarily on binary decisions and which form the basis of decisions ranging from special education provision in schools to reimbursement from health insurance companies [55]. Our findings suggest that a dimensional approach based on severity of distress, rather than individual symptoms, should be more explicit and central to clinical practice and policy. For patients, such an approach could help explain diagnostic fluctuations over the life course and allow for the tailoring of interventions to focus not on a single diagnosis but rather on an individual’s specific vulnerabilities and processes [11].

### Limitations

This study is fundamentally limited by the range of symptoms considered: NSPN includes 83 items about negative phenomena and 24 about positive phenomena; ALSPAC includes 66 items about negative phenomena and no items about positive phenomena. The inclusion of additional items may lead to the discovery of additional classes, or even differential classification altogether. However, the items included do address the most prevalent negative phenomena (symptoms) [1] and the positive phenomena are relevant to all individuals. A related issue within the category of negative phenomena is the under-representation of externalizing symptoms. Indeed, there was only one externalizing measure available in one of two cohorts. Future studies need to address this limitation by exploring whether increasing the breadth of externalizing symptoms included in the analyses significantly alters the results. We were further limited by the availability of data against which to validate our findings. Recognising the limitations of traditional diagnostic categories, it would have been preferable to characterise the classes using other clinical endpoints, such as psychosocial functioning or treatment utilisation. However, data on these were not available. Finally, there were two technical limitations: first, the EM algorithm would not converge when fitting the VEI model with five or more classes. Therefore, it is possible that a VEI model with five or more classes could fit better than our solutions. However, it is typical for such non-convergence to be caused by the existence of very similar classes that are often generated by models seeking high numbers of classes. This leads to singularity of the covariance matrix [38] and estimation problems. Second, most of measures used in this study are screening scales and therefore some items show floor or ceiling effects. It is known that some classification methods may form spurious classes in such situations [56]. To our knowledge, however, there is no literature showing how severely skewed distributions of scale items may affect GMM results. This is a serious gap in the literature, but is outside of scope of this study.

## Conclusions

In conclusion, our results show that classes of mental phenomena in the adolescent population may differ according to severity of transdiagnostic distress. Current diagnostic conceptualizations of psychopathology may need to be revised in light of empirical findings, and a dimensional rather than categorical approach may be warranted.

## Supplementary Information

Below is the link to the electronic supplementary material.

Supplementary file1 (PDF 589 KB)

## Data Availability

Not applicable.
