# Supplementary material for: Stratification of adolescents across mental phenomena emphasizes the importance of transdiagnostic distress: a replication in two general population cohorts
Source: Eur Child Adolesc Psychiatry. 2021 Nov 18;32(5):797–807. doi: 10.1007/s00787-021-01909-0 (PMC10147756; doi:10.1007/s00787-021-01909-0)
Supplement: Supplementary file 1 — Supplementary file1 (PDF 589 KB) [file 787_2021_1909_MOESM1_ESM.pdf]

TableS1: Measures and their descriptive statistics

Study	Measure	Domain	Description	Mean	SD	Median	Min	Max	Reliability (Cronbach's alpha
NSPN	33-item Moods and Feelings Questionnaire (MFQ) (Costello & Angold, 1988)	Depression	Four-category ('never', 'mostly', 'sometimes', and 'always'); Self report, previous fortnight; all items negatively worded	18.41	13.48	15	0	87	0.94
	28-item Revised Children's Manifest Anxiety Scale (RCMAS) (Reynolds & Richmond, 1978)	Generalized anxiety	Four-category ('never', 'mostly', 'sometimes', and 'always'); Self report, previous fortnight; all items negatively worded	19.03	14.48	16	0	84	0.95
	11-item Revised Leyton Obsessional Inventory (LOI) (Bamber, Tamplin, Park, Kyte, & Goodyer, 2002)- compulsion subscale	Obsessive compulsiveness	Four-category ('never', 'mostly', 'sometimes', and 'always'); Self report, previous fortnight; all items negatively worded	0.80	1.76	0	0	12	0.78
	11-item Revised Leyton Obsessional Inventory (LOI) (Bamber et al., 2002) – obsession subscale	Obsessive compulsiveness	Four-category ('never', 'mostly', 'sometimes', and 'always'); Self report, previous fortnight; all items negatively worded	2.29	2.31	2	0	12	0.73
	11-item Revised Leyton Obsessional Inventory (LOI) (Bamber et al., 2002)- cleanliness subscale	Obsessive compulsiveness	Four-category ('never', 'mostly', 'sometimes', and 'always'); Self report, previous fortnight; all items negatively worded	1.71	2.09	1	0	9	0.76
	11-item Antisocial Behaviour Questionnaire (ABQ)	Anti-social behaviors (violating social norms, destructive behaviors, violence to people, lying, and stealing)	Four-category ('never', 'mostly', 'sometimes', and 'always'); Self report, previous fortnight; all items negatively worded	1.26	2.09	1	0	24	0.74
	10-item Rosenberg Self-Esteem Scale (RSES) (Rosenberg, 1965)	Self-esteem	Four-category ('never', 'mostly', 'sometimes', and 'always'); Self report, previous fortnight; 5 items positively worded (remainder negatively worded); negatively worded items in RSES were reversed to obtain interpretable RSES scores	19.60	6.37	20	0	30	0.91
	14-item Warwick-Edinburgh Mental Wellbeing Scale (WEMWBS) (Tennant et al., 2007)	Mental wellbeing (positive evidence of current happiness, personal activity, and personal achievement)	Five-category ('none of the time', 'rarely', 'some of the time', 'often', 'all of the time'), with higher scores indicated greater mental wellbeing; Self report, previous fortnight; all items positively worded	48.60	9.56	50	16	70	0.92

Study	Measure			Mean	SD	Median	Min	Max	Reliability (Cronbach's alpha
ALSPAC	13-item short version of MFQ (SMFQ) (Angold et al., 1995)	Depression	Three-category ('not true', 'sometimes true', 'true')	18.67	5.46	17	13	39	0.91
	32 items[a] from Development and Well Being Assessment (DAWBA) (Goodman, Ford, Richards, Gatward, & Meltzer, 2000)	Anxiety	DAWBA items are scored on a three-point scale with varying wording of response categories, but the lowest category always indicates absence of symptom, the middle category irregular presence and the highest category more regular occurrence; Assessed at age 15.5 years	28.85	5.35	27	23	60	0.80
	10 items from Psychosis-Like Symptoms Questionnaire (PLIKS-Q) (Zammit, Owen, Evans, Heron, & Lewis, 2011)	Psychotic experiences	Four-point scale ('no, never', 'yes, sometimes', 'yes, often', 'nearly always')	10.93	1.82	10	10	30	0.74
	11 negative symptoms from Community Assessment of Psychic Experiences (CAPE) (Stefanis et al., 2002)	Negative symptoms	Three-point scale ('no, never', 'yes, maybe', 'yes, definitely')	17.64	5.51	16	11	44	0.88

[a] 35 items relating to generalized anxiety disorder (GAD), agoraphobia, specific phobias, and post-traumatic stress disorder (PTSD) were selected from the DAWBA. "Gated" item pairs, where answering "yes" to an initial question lead onto an additional question, were collapsed into single items resulting in 32 items used for analysis (as described in Jones et al.[14]).

FigS1: ICLs of fitted Gaussian mixture models for NSPN (left) and ALSPAC (right). Missing points for some models indicate that the specific model did not converge. Model acronyms are explained in Table 1 and detailed in Fraley et al. (2012).

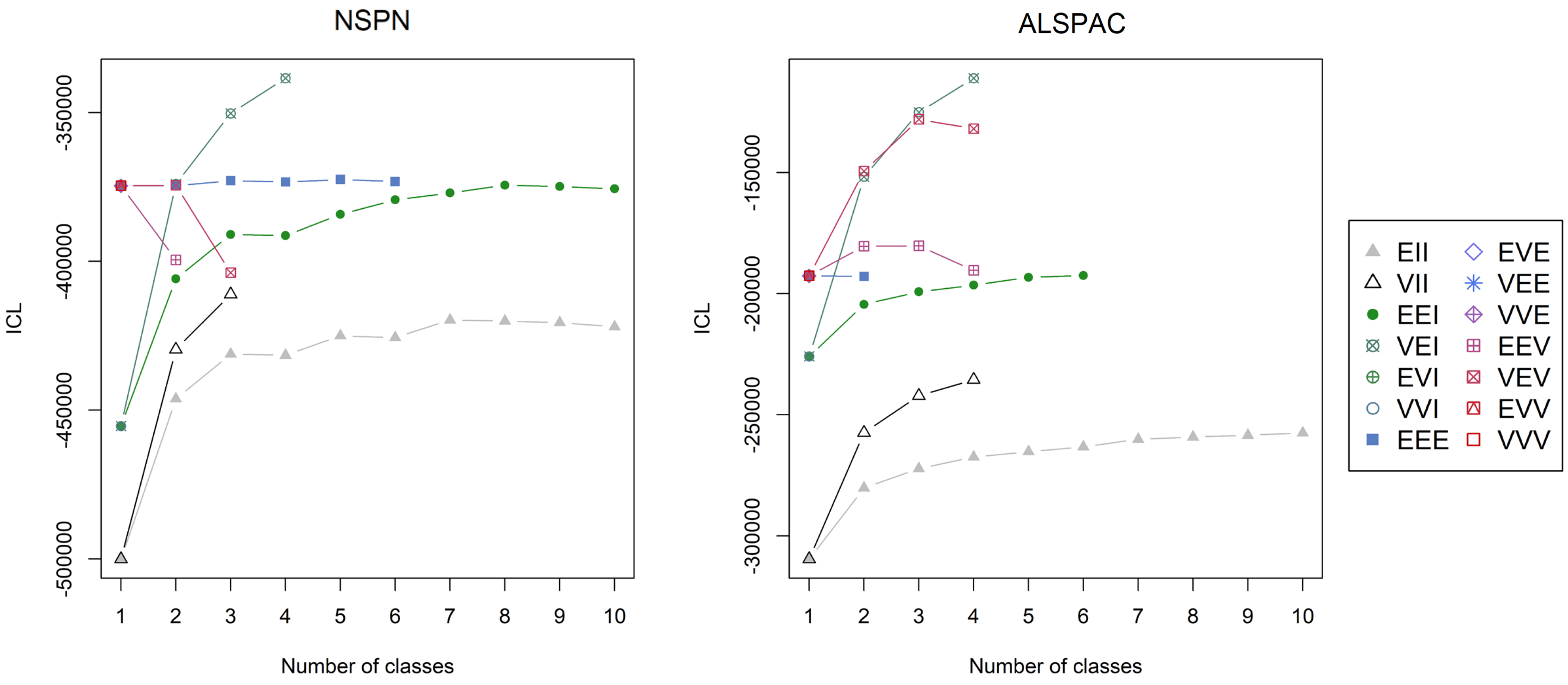

FigS2: Entropy and number of clusters for NSPN (left) ALSPAC (right). The x-axes are rescaled (to show cumulative count of individuals across classes) to address the different number of individuals in each cluster. The dashed line represents the fitted piecewise regression line.

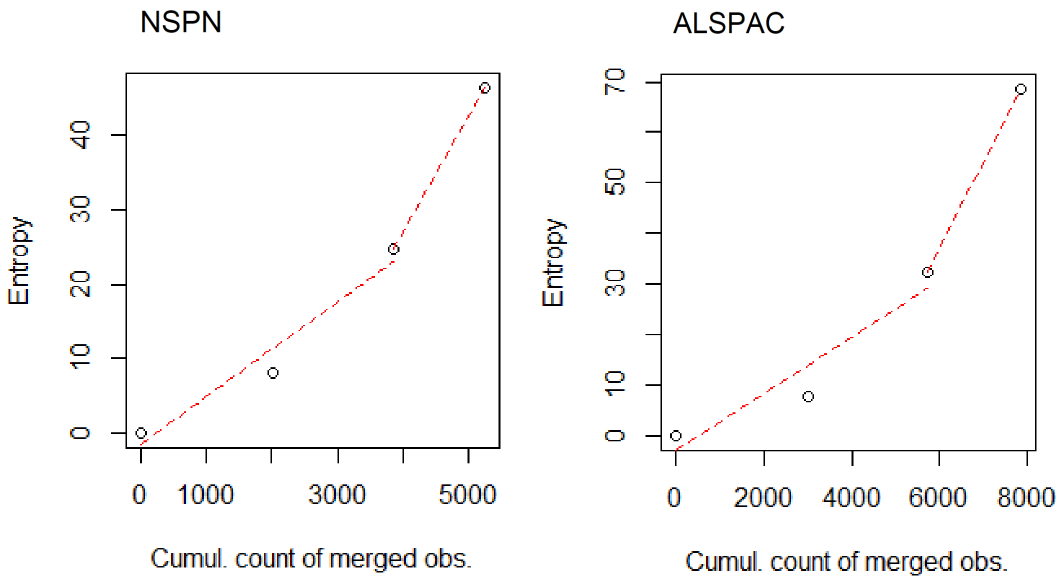

FigS3: Simplified 3D plot of distances between individuals within NSPN (top) and ALSPAC (bottom). Colours represent different classes. Axes represent the first three principal components of the distance matrix (x=PC1, y=PC2, z=PC3)

NSPN

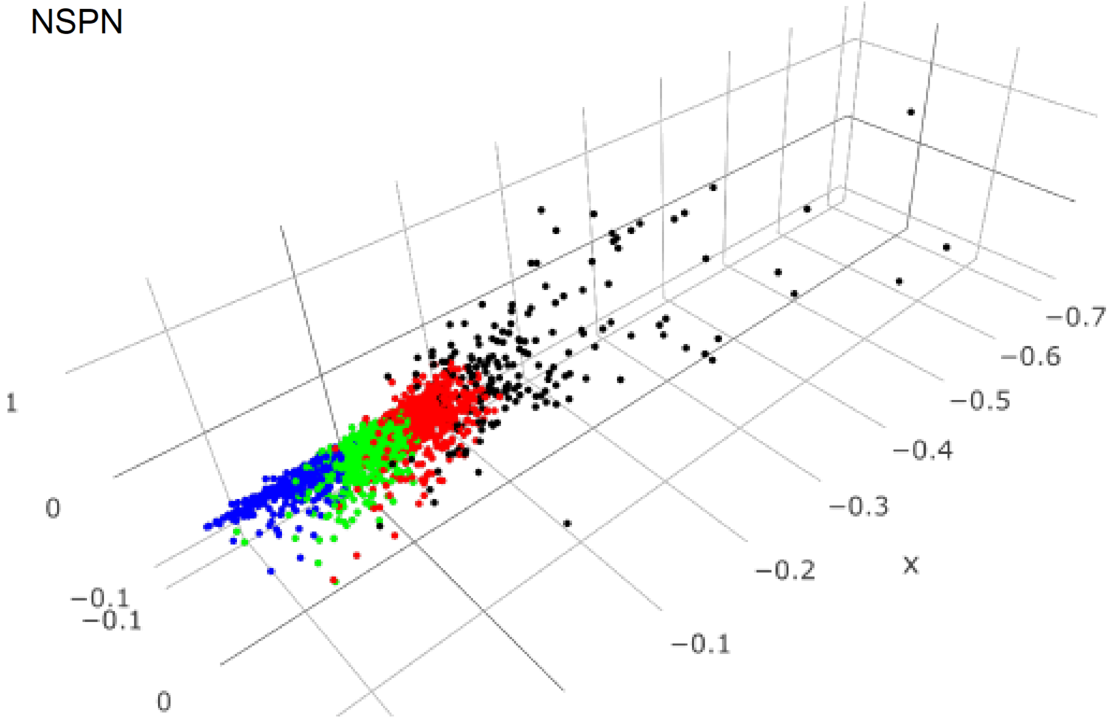

ALSPAC

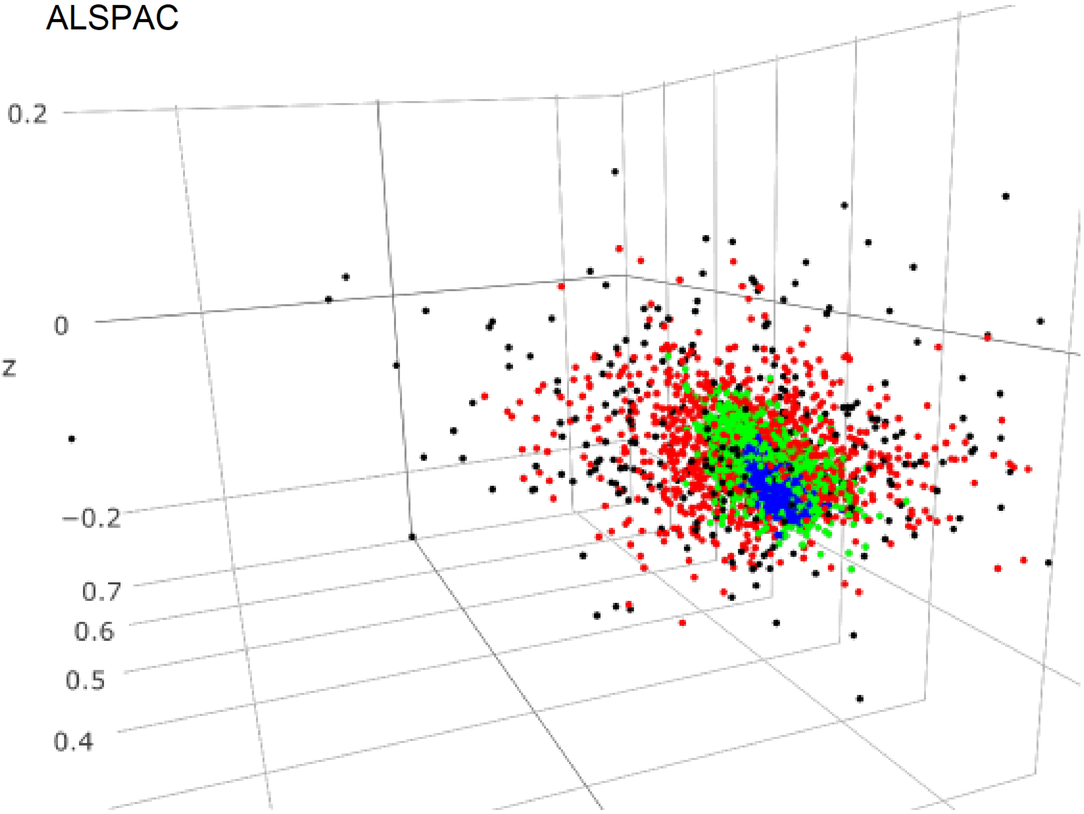

References

Angold, A., Costello, E. J., Messer, S. C., Pickles, A., Winder, F., & Silver, D. (1995). The development of a short questionnaire for use in epidemiological studies of depression in children and adolescents. *International Journal of Methods in Psychiatric Research, 5*, 237-249.

Bamber, D., Tamplin, A., Park, R. J., Kyte, Z. A., & Goodyer, I. M. (2002). Development of a short leyton obsessional inventory for children and adolescents. *Journal of the American Academy of Child and Adolescent Psychiatry, 41*(10), 1246-1252.

Costello, E., & Angold, A. (1988). Scales to Assess Child and Adolescent Depression: Checklists, Screens, and Nets. *Journal of the American Academy of Child & Adolescent Psychiatry, 27*(6), 726-737.

Fraley, C., Raftery, A. E., Murphy, T. B., & Scrucca, L. (2012). mclust Version 4 for R: Normal Mixture Modeling for Model-Based Clustering, Classification, and Density Estimation.

Goodman, R., Ford, T., Richards, H., Gatward, R., & Meltzer, H. (2000). The Development and Well-Being Assessment: description and initial validation of an integrated assessment of child and adolescent psychopathology. *Journal of Child Psychology and Psychiatry, 41*(5), 645-655.

Jones, H. J., Heron, J., Hammerton, G., Stochl, J., Jones, P. B., Cannon, M., et al. (2018). Investigating the genetic architecture of general and specific psychopathology in adolescence. *Translational Psychiatry, 8*(1), 145.

Reynolds, C. R., & Richmond, B. O. (1978). What I think and feel: A revised measure of children's manifest anxiety. *Journal of Abnormal Child Psychology, 6*(2), 271-280.

Rosenberg, M. (1965). *Society and the adolescent self-image* (Vol. 11): Princeton university press Princeton, NJ.

Stefanis, N. C., Hanssen, M., Smirnis, N. K., Avramopoulos, D. A., Evdokimidis, I., Stefanis, C. N., et al. (2002). Evidence that three dimensions of psychosis have a distribution in the general population. *Psychological Medicine, 32*(02), 347-358.

Tennant, R., Hiller, L., Fishwick, R., Platt, S., Joseph, S., Weich, S., et al. (2007). The Warwick-Edinburgh Mental Well-being Scale (WEMWBS): development and UK validation. *Health and Quality of Life Outcomes, 5*, 63.

Zammit, S., Owen, M. J., Evans, J., Heron, J., & Lewis, G. (2011). Cannabis, COMT and psychotic experiences. *British Journal of Psychiatry, 199*(5), 380-385.
